# Decoupling microbial iron reduction from anoxic microsite formation in oxic sediments: a microscale investigation through microfluidic models

**DOI:** 10.3389/fmicb.2025.1504111

**Published:** 2025-01-28

**Authors:** Giulia Ceriotti, Alice Bosco-Santos, Sergey M. Borisov, Jasmine S. Berg

**Affiliations:** ^1^Faculty of Geoscience and Environment, Institute of Earth Surface Dynamics, University of Lausanne, Lausanne, Switzerland; ^2^Faculty of Technical Chemistry, Chemical and Process Engineering and Biotechnology, Institute of Analytical Chemistry and Food Chemistry, Graz University of Technology, Graz, Austria

**Keywords:** iron reduction, oxygen, microfluidics, planar sensors, diffusion, *Shewanella oneidensis*, anoxic microsites, iron cycle

## Abstract

Iron (Fe) reduction is one of the oldest microbial processes on Earth. After the atmosphere and ocean became oxygenated, this anaerobic process was relegated to niche anoxic environments. However, evidence of Fe reduction in oxic, partially saturated subsurface systems, such as soils and vadose zones, has been reported, with the common explanation being the formation of anoxic microsites that remain undetected by bulk measurements. To explore how microscale oxygen concentrations regulate microbial Fe reduction, we cultivated a facultative Fe-reducing bacterium using a microfluidic setup integrated with transparent planar oxygen sensors. Contrary to expectations, Fe reduction occurred under fully oxic conditions, without the formation of anoxic microsites. Our results suggest that microbially mediated Fe-reduction could be more widespread in oxic subsurface environments than previously assumed. Moreover, our mathematical modeling of oxygen dynamics around biomass-rich layers revealed that the onset of anoxia is mainly controlled by biomass spatial organization rather than the conventionally used water saturation index. This opens a new perspective on the proxies needed to predict anoxic microsite formation and Fe(III) reduction occurrence.

## 1 Introduction

Iron (Fe) is one of the major elements in the Earth’s crust and microorganisms have been cycling it for at least 3.0 billion years (Lyons et al., 2024) between its oxidized ferric [Fe(III)] and more soluble reduced ferrous [Fe(II)] forms. Today, most of the Fe on the planet’s surface is found within minerals such as Fe(III) oxides in rocks (Posth et al., 2011), sediments (Gankhurel et al., 2022), and soils (Malakar et al., 2022). Due to their high adsorbent capacity, Fe(III) oxides function as nutrient and metal scavengers in these environments. Thus, reductive dissolution of Fe-oxides regulates the mobility and bioavailability of adsorbed metals and nutrients. In other words, Fe reduction drives crucial environmental processes such as soil organic matter turnover and contaminant fate in the subsurface (Royer et al., 2002).

A complex network of abiotic and biotic reactions contributes to the cycling of Fe between redox states. Facultative metal-reducing bacteria (Abboud et al., 2005) are key players in such a network, capable of anaerobically respiring Fe(III) and releasing bioavailable Fe(II) into the environment. Facultative Fe-reducers are assumed to preferentially respire molecular oxygen (O_2_) according to the established thermodynamic electron acceptor cascade (Jørgensen, 2000). Therefore, like all anaerobic metabolisms, facultative microbial Fe(III) reduction is thought to be confined to anoxic (O_2_-depleted) environments. Nonetheless, several studies have measured Fe(II) as free ions or complexed with organic matter in oxic natural subsurface systems, e.g., partially saturated soils (Kügler et al., 2019; Malakar et al., 2020; Daugherty et al., 2017), and experimental setups (Arnold et al., 1990; Swanner et al., 2018; Huang et al., 2021; Malakar et al., 2020; Küsel et al., 2002; Lueder et al., 2022), suggesting the occurrence of Fe(III) reduction despite prevailing oxidizing conditions.

The observation of microbial Fe(III) reduction in oxic environments is usually explained by the formation of anoxic microsites—microscopic O_2_-depleted zones within otherwise oxic sediments- where facultative Fe(III) reducers switch to anaerobic respiration. Such microhabitat formation is driven by intense aerobic respiration by concentrated microbial biomass, such as in colonies, biofilms, or liquid layers densely populated by planktonic cells. These bacterial aggregates create localized hotspots of intense O_2_ consumption via aerobic respiration that outpaces the slow diffusive O_2_ supply in water (Malakar et al., 2020; Or et al., 2007; Keiluweit et al., 2018; Baveye et al., 2018; Ceriotti et al., 2022).

Organic matter (OM) compounds, commonly found in natural environments, are known to form complexes with Fe(II). Depending on the molecular structure of the organic ligand, OM-Fe(II) complex formation can accelerate or retard Fe(II) re-oxidation by molecular O_2_ (Daugherty et al., 2017; Rose and Waite, 2002). For example, Daugherty et al. (2017) showed that a model humic acid significantly slows Fe(II) oxidation by O_2_, and the presence of OM can even trigger a dynamic redox cycle capable of maintaining stable Fe(II) concentrations in solution despite the presence of O_2_. As a result, Fe(II) produced in anoxic microsites can persist and accumulate in water despite the presence of O_2_.

**FIGURE 1:**
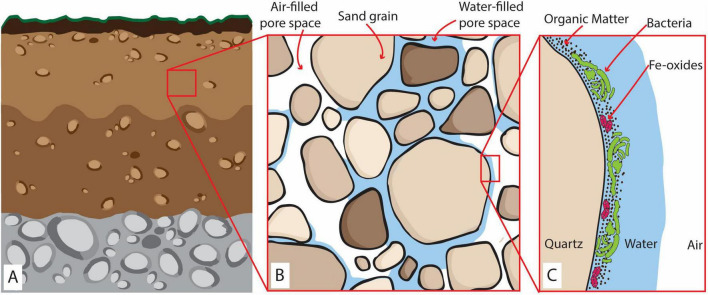
**(A)** Representation of a partially saturated oxic subsurface environment (e.g., soils and vadose zones). **(B)** The zoom-in at the millimeter scale shows the coexistence of sand grains, air-filled, and water-saturated pores. **(C)** The zoom-in at the microscale represents biomass distribution and accumulation of OM and Fe-oxides within the water layer covering the grain surface surrounded by air-rich pore space. Here, four layers can be identified: (i) an air layer, constituting the constant source of O_2_; (ii) a water layer of variable thickness depending on the saturation of the sediments; (iii) an OM-Fe-rich film where the microbes will grow creating a biomass-rich layer and (iv) the solid surface of the sand grain (quartz) forming an impermeable layer.

The presence of organic matter along with microbial aerobic respiration, Fe reducers, and Fe(III)-oxides under O_2_ diffusion-dominated conditions is expected in many natural environments, notably in oxic soils and vadose zones, as exemplified in Figure 1. These environments thus create favorable conditions for the formation of anoxic microsites, promoting microbial Fe(III) reduction and Fe(II) accumulation. Due to their small size and ephemeral lifetime of hours to days (Lacroix et al., 2023), anoxic microsites are challenging to detect and monitor using traditional O_2_ measurements that capture only the bulk conditions of a system (Keiluweit et al., 2018; Baveye et al., 2018). Therefore, the correlation between microbial Fe(III)-oxide reduction and anoxic microsite formation remains unclear. To elucidate the mechanisms of anoxic microsite formation and quantify their relevance for microbial Fe(III) reduction in a bulk oxic environment, it is imperative to simultaneously map O_2_ concentrations at the microscale and assess the occurrence of Fe(III) reduction.

The recent development of sensing microfluidic devices—microfluidic chips integrated with transparent luminescent planar sensors—has enabled the simultaneous mapping of O_2_, opaque objects (such as minerals), and biomass in real-time at the microbial (micron)scale. Originally developed for biomedical applications (Ungerböck and Mayr, 2018; Sun et al., 2015; Ungerböck et al., 2013), these sensing devices have provided unprecedented insights into microscale biogeochemical dynamics in soils and sediments (Ceriotti et al., 2022; Borer et al., 2018). We designed an O_2_-sensing microfluidic reactor to grow the common facultative Fe-reducer, *Shewanella oneidensis* MR-1, with poorly crystalline Fe(III) oxides (ferrihydrite) and OM for a few days. Our experiment simulated the concurrence of aerobic biomass growth and Fe reduction in the presence of Fe(III)-oxides and OM in an oxic, diffusion-dominated environment. In other words, we mimicked the conditions on a sand grain surface in partially saturated sediment (see Figure 1C) in a simplified and replicable way. Combining microfluidic reactor experiments with photometric Fe(II) measurements, we could relate biomass, ferrihydrite, and O_2_ concentration distribution at the microscale to Fe reduction in the system. Finally, our results were used to construct a mathematical model exploring the extent to which water layer thickness controls O_2_ balance and the onset of anoxic conditions within O_2_ diffusion-dominated systems.

## 2 Materials and methods

### 2.1 Experimental setup

#### 2.1.1 Bacterial culture

*Shewanella oneidensis* MR-1 (*S. oneidensis*) is among the most well-studied facultative Fe-reducing bacteria isolated from natural lake sediments (Abboud et al., 2005; Shi et al., 2012). *S. oneidensis* inoculum was grown from frozen stocks overnight under oxic conditions in 5 mL of Luria-Bertani (LB, Sigma Aldrich) broth at 30°C in an orbital shaker (180 rpm).

#### 2.1.2 O_2_-sensing microfluidic reactor

**FIGURE 2:**
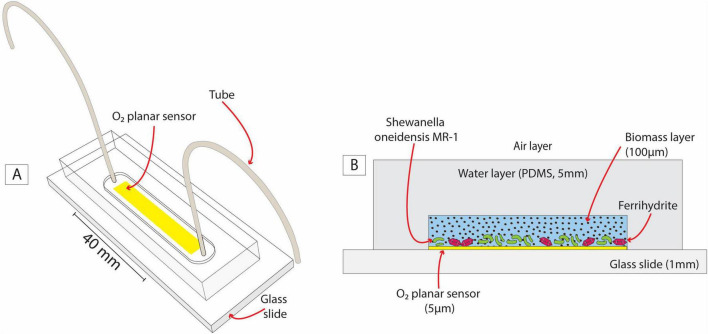
A sketch of the O_2_-sensing microfluidic reactor **(A)** and its cross-section **(B)**. The latter highlights the analogy between the microfluidic setup and sediment grain in a partially saturated system with a layer of biomass, water, and air.

Using a silica mold prepared with classical soft lithography, we produced a microfluidic reactor shaped as a straight channel (Figure 2A) of 40 mm (l) × 4.5 mm (w) × 100 μm (h) (a total volume of ∼ 18 mL, microfluidic fabrication and assembly details are provided in Supplementary material – section 1.1) engraved into a 5 mm thick polydimethylsiloxane polymer structure (PMDS, Sylgard 184 Silicone Elastomer mixed with 10 w/w % of curing agent; supplier: Dow Corning, Midland, MI). Two Tygon tubes (Cole-Palmer, inner/outer diameter 0.02/0.04 inches) connected at the ends of the channel completed the microfluidic reactor to allow inoculation as detailed below in the incubation setup. The reactor was integrated with a non-invasive transparent O_2_ planar sensor screen-printed on the glass surface before microfluidic reactor assemblage. These sensors are thin films (∼5 μm) composed of a mixture of two luminescent dyes immobilized into a polymeric matrix (polystyrene) covering a surface of 26 mm (l) × 4 mm (w) (Ceriotti et al., 2022). Both dyes are excited at the same wavelength (450 nm) but emit two distinguishable luminescent signals: one is peaked at 650 nm and is quenched by O_2_; the second dye provides a reference signal peaked at 500 nm (see sections 1.2 in Supplementary material for details). Previous studies showed that these sensors, integrated into microfluidic devices, successfully detected microscale O_2_ gradients that are undetectable by bulk measurements (Ungerböck and Mayr, 2018; Sun et al., 2015; Ungerböck et al., 2013; Ceriotti et al., 2022).

#### 2.1.3 Incubation setup

After sterilization and degassing (see Supplementary material—section 1.1), the microfluidic reactor was saturated with a freshly prepared medium (20 mM PIPES, 2 mM ferrihydrite and 10X LB) with in-house synthesized Fe-oxides (Supplementary material—section 2) inoculated with the *S. oneidensis* overnight culture (1:50 v/v, corresponding to an OD_600_ of 0.03). A negative control experiment was conducted using only the sterile medium. The use of LB, mostly composed of yeast extract, as a source of electron donors and nutrients aims to mimic the complexity of carbon sources often encountered in natural systems (e.g., marshlands, peat bogs, eutrophic lakes, soils, and even in and around marine snow), as previously suggested in the literature (Zhang et al., 2021). Three replicates were performed for each condition. After saturation, the tube extremities were connected to a sterile 0.22 μm filter and immersed in an Erlenmeyer flask filled with sterile diluted LB broth in deionized water (1:10 v/v, see Supplementary material—section 1.1 for details) to maintain the channel saturated for 72 h under no-flow conditions.

The purpose of the microfluidic reactor (Figure 2) was to mimic the growth of aerobic and Fe-reducing biomass in the presence of OM and Fe-oxides in the subsurface (e.g., Figure 1C) under replicable and controlled conditions. The glass slide acted as an impermeable *solid surface*, like sand grains. A 100 μm-aqueous layer enriched with OM and Fe-oxides (ferrihydrite) favored biomass growth and will be referred to as the *biomass layer* in the following. The gas-permeable layer (PDMS) ensured a continuous O_2_ supply by diffusion from the surrounding air. The PDMS and pure water present very similar O_2_ diffusion coefficients (3.2 × 10^–9^ m^2^ s^–1^ and 2 × 10^–9^ m^2^ s^–1^, respectively) (Markov et al., 2014). Therefore, from the point of view of the system’s O_2_ balance, we assume that the PDMS layer mimicked a *water layer* overlying the *biomass layer*, connecting it to the *air layer*, as outlined in Figure 2B.

In this system design, *S. oneidensis* can respire and grow aerobically, forming a biomass-rich layer. Simultaneously, under suitable microscale conditions (i.e., anoxic microsites), *S. oneidensis* can switch to facultative anaerobic respiration, triggering Fe(III) reduction. The PDMS walls are impermeable to solutes, thus retaining in the liquid phase any Fe(II) produced for measurement described in section 2.2.2. The O_2_ planar sensor was positioned at the interface between the solid surface and the *biomass layer*, where anoxic microsites are most likely to form due to the combined effect of aerobic respiration and the limited O_2_ diffusion imposed by the solid surface.

### 2.2 Data acquisition

#### 2.2.1 Biomass and O_2_ maps

To monitor O_2_ concentration in real-time at the microscale in parallel with bacterial growth, an automated microscope (inverted Nikon Eclipse Ti-E2) equipped with a 10X objective and a CMOS scientific camera (DS-Qi2 Nikon) took pictures (16-bit black and white, 0.29 μm/pixel) of ∼60% of the channel surface every hour for 72 h. The microscope switched between different optical configurations: bright field, adjusted phase contrast, and two fluorescence configurations. Collected pictures were post-processed to map (a) ferrihydrite grains, (b) biomass organization, and (c) O_2_ concentrations. Technical details about the setup of the optical configurations and image processing are in Supplementary material (sections 3 and 4).

#### 2.2.2 Fe(II) concentrations

We conducted five identical microfluidic incubations in order to measure Fe(II) concentrations in microfluidic reactors. This allowed us to extract and mix the medium from the five channels, providing a sufficient liquid volume (>70 μL) for Fe(II) spectrophotometric measurements using the Ferrozine assay (Stookey, 1970; see Supplementary material—section 5 for details on method and calibration). This procedure was repeated to obtain three independent Fe(II) measurements (i) for the initial conditions, (ii) after 72 h of *S. oneidensis* incubation, and (iii) of sterile medium incubation (negative control). All microfluidic reactors were kept in strict darkness to prevent light-induced abiotic Fe(III) reduction.

## 3 Experimental results

**FIGURE 3:**
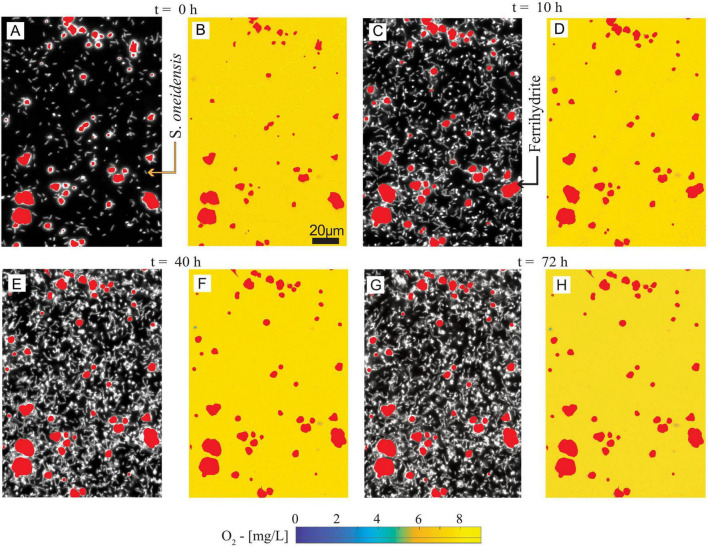
**(A,C,E,G)** Spatial distribution of ferrihydrite and *S. oneidensis* at *t* = 0, 10, 40, and 72 h. **(B,D,F,H)** Spatial distribution of O_2_ concentration and ferrihydrite at *t* = 0, 10, 40, and 72 h, corresponding to **(A,C,E,G)**, respectively. The maps have a 0.29 μm/pixel resolution, or ∼1/6th of a bacterial cell length.

**FIGURE 4:**
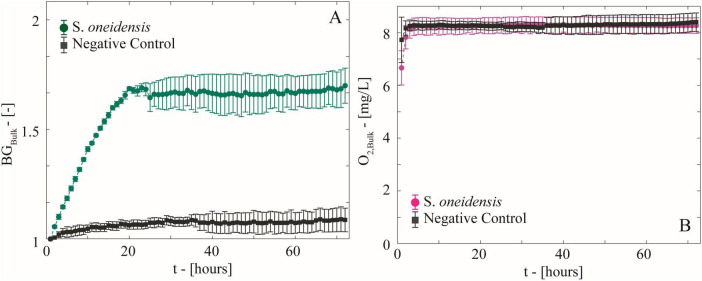
**(A)** Temporal behavior of biomass growth (BG_Bulk_) measured as the average increment in light diffraction normalized to the initial value in the microfluidic reactors with growing *S. oneidensis* and the negative control. **(B)** Temporal behavior of bulk O_2_ concentration computed as the average of spatial O_2_ concentration maps.

S. *oneidensis* was grown on diluted LB with ferrihydrite in microfluidic reactors to simulate, in a simplified and replicable manner, the growth of biomass and the formation of anoxic microsites on sand grain surfaces in partially saturated subsurface environments (Figures 1C, 2B). The real-time maps of biomass, ferrihydrite, and O_2_ concentration have been post-processed to analyze both microscale biomass and O_2_ spatial distribution (Figure 3), and bulk-scale (Figure 4) biomass growth (BG_Bulk_) and bulk O_2_ concentrations (O_2,Bulk_).

### 3.1 Oxic conditions persisted in *Shewanella* incubations

Biomass maps revealed an increasingly populated system (Figures 3A,C,E,G). Biomass growth (BG_Bulk_) at the bulk level (Figure 4A), followed a fast-growing trend within the first 24 h, consistent with the aerobic lifestyle of *S. oneidensis*. This was followed by a stationary phase of stable biomass content. Bulk O_2_ concentrations (Figure 4B), obtained by averaging the hourly imaged O_2_ concentration maps (e.g., Figures 3B,D,F,H), revealed a negligible impact of aerobic microbial respiration on the overall O_2_ balance during both the rapid growth and stationary growth phases of *S. oneidensis*. In fact, bulk O_2_ concentrations remained close to saturation and were indistinguishable from the negative control. In other words, bulk oxic conditions persisted in the system.

### 3.2 No anoxic microsites were formed during *S. oneidensis* biomass-rich layer formation in the microfluidic reactor

Our reactor was colonized by uniformly distributed biomass (Figures 3A,C,E,G). Oxygen concentration maps (Figures 3B,D,F,H) were processed to detect the formation of anoxic microsites by identifying the portions of the reactor space characterized by microoxic/anoxic conditions, i.e., an O_2_ concentration ≤ 0.32 mg L^–1^ (Berg et al., 2022; see Supplementary material—section 6 for procedures and data). No anoxic microsites were detected in the system during our measurements, with oxic conditions (>4 mg L^–1^) persisting at the microscale across the colonized solid surface. After 20 h from the beginning of the experiment, the O_2_ concentrations were persistently and coherently stable at both the bulk and microscale until the end of the experiment, when Fe(II) was measured.

### 3.3 Fe(II) was produced and accumulated under fully oxic conditions

**FIGURE 5:**
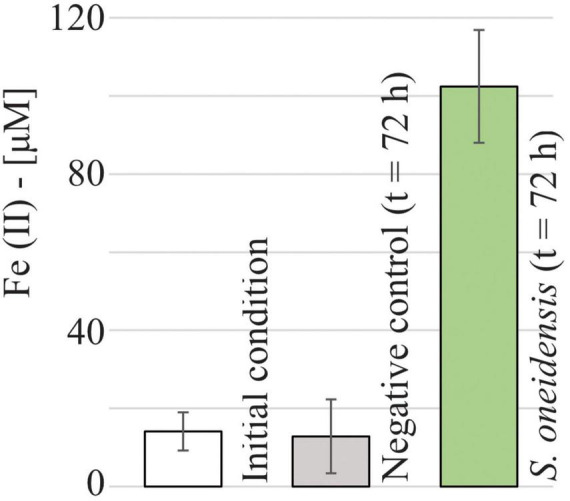
Measured Fe(II) concentrations at *t* = 0 h (14.1 ± 4.9 μM) and after 72 h of sterile medium incubation (negative control, 12.8 ± 9.4 μM) and *S. oneidensis* incubation (102.5 ± 14.4 μM) in the microfluidic reactor.

Despite the absence of anoxic microsites in our system, a high concentration of dissolved Fe(II) (102.5 ± 14.4 μM, Figure 5) was detected in the microfluidic reactor inoculated with *S. oneidensis* after 72 h of incubation, showing a net increase (Δ = 88.4 ± 19.3 μM) compared to the initial Fe(II) concentration (14.1 ± 4.9 μM). In contrast, Fe(II) concentrations in the negative control remained indistinguishable from the initial conditions. In other words, Fe(III) reduction occurred under fully oxic conditions in the presence of live *S. oneidensis*.

## 4 Discussion

### 4.1 Ferrihydrite reduction occurs under oxic conditions

The significant production of Fe(II) in biotic but not abiotic incubations suggests that *S. oneidensis* growth contributes to ferrihydrite reduction. Surprisingly, Fe reduction occurred under fully oxic conditions, as confirmed by our planar oxygen sensor measurements. The absence of anoxic microsite formation here contrasts with previous microfluidic experiments using the same O_2_ sensing technique to detect the formation of anoxic microsites induced by a *Pseudomonas* strain (Ceriotti et al., 2022). This difference can be primarily explained by the higher gas-permeability of our microfluidic setup along with the absence of tortuous porous structures and flow-induced shear stress that favored O_2_ diffusion at the microscale (see section 1.3 of Supplementary material for further explanation).

Traditionally, facultative Fe-reducing bacteria are thought to utilize Fe(III) as a terminal electron acceptor only in the absence of O_2,_ which has a higher redox potential (Beliaev et al., 2005; Fredrickson et al., 2008). The limit at which the terminal respiratory enzymes of *S. oneidensis* remain active has been measured as low as 0.03 mg/L (Le Laz et al., 2014; Bertling et al., 2021; Pitcher and Watmough, 2004; Zhou et al., 2013). However, the critical point at which these bacteria switch from aerobic to anaerobic respiration may not be as clear-cut as commonly assumed. Microbial reduction of Fe(III) in the presence of O_2_ was suggested as early as the 1990s for *Shewanella putrefaciens* sp. strain 200 (Arnold et al., 1990) and has also been proposed for Actinobacteria (Zhang et al., 2019). For example, the stepwise addition of the aerobic respiration inhibitor CN^–^ to oxic cultures of *S. putrefaciens* led to a gradual reduction in O_2_ utilization without stopping Fe(III) reduction (Arnold et al., 1990). *S. putrefaciens* was thus shown to perform Fe(III) reduction and aerobic respiration simultaneously. Nonetheless, the O_2_-measurements employed in these previous works were limited to bulk concentrations, which could not rule out the development of anoxic microsites at the microscale, i.e., the scale relevant to microbes. Our experimental design overcomes this technological limitation and confirms the occurrence of dissimilatory Fe(III) reduction under fully oxic conditions at the microscale.

Elucidating the physiological mechanism behind Fe(III) reduction would require detailed studies of genetic expression and enzymatic activity, which are beyond the scope of our current research. Nonetheless, we can speculate about the reasons behind oxic Fe(III) reduction. For one, *S. oneidensis* may retain the ability to respire both O_2_ and Fe(III) at the population or cellular level. This ecological strategy is known for facultative denitrifiers, which maintain enzymatic machinery for both aerobic and anaerobic respiration to adapt to fluctuating redox conditions (Marchant et al., 2017).

It is also possible that *S. oneidensis* indirectly mediates Fe(III) reduction via electron shuttles, such as endogenously produced riboflavin, without direct cell-mineral contact. Experimental evidence shows that, under anoxic conditions, *S. oneidensis* produces reduced riboflavin to transfer electrons to iron minerals, which act as the final electron acceptor (Kotloski and Gralnick, 2013). Under oxic conditions, reduced riboflavins are rapidly re-oxidized by O_2_ and might be a less efficient electron shuttling mechanism. Interestingly, *S. oneidensis* still and more abundantly produces endogenous riboflavins in the presence of O_2_ (Kim et al., 2016), which are known to participate in complex redox reactions involving both Fe(II) and Fe(III)-complexes and reactive O_2_ species (Chen et al., 2023). This reaction network could mediate Fe reduction in oxic conditions, albeit through mechanisms possibly distinct from those in anoxic environments.

Iron reducers are also able to use exogenous soluble redox-active molecules, like quinone moieties, typically abundant in natural OM, as electron shuttles to reduce Fe-oxides (Royer et al., 2002; Royer et al., 2002; Bauer and Kappler, 2009). Indeed, the presence of OM enhances microbial Fe reduction under anoxic conditions (Royer et al., 2002; Royer et al., 2002). Bauer and Kappler (2009) showed that some OM compounds retain reduced functional groups that are not oxidized by O_2_ but can be abiotically oxidized by Fe(III) present within poorly crystalline Fe-oxides like ferrihydrite. These OM functional groups could serve as electron acceptors for *S. oneidensis* and then be re-oxidized by ferrihydrite, liberating new electron-accepting sites in a continuous redox cycle. In other words, OM could enable indirect microbially mediated ferrihydrite reduction under fully oxic conditions. In agreement with these findings, Daugherty et al. (2017) reported that reduced quinones, often contained in natural organic matter, can reduce Fe(III) to Fe(II) at a rate that can outcompete Fe(II) oxidation by molecular O_2_ or reactive oxygen species. In our setup, LB broth, mostly composed of yeast extract, consists of many redox-active molecules (Shlosberg et al., 2022) and mimics the complexity of natural OM, creating favorable conditions for indirect microbially mediated Fe(III) reduction via electron shuttling. Similarly, the lysis of dead *S. oneidensis* cells could contribute as a source of electron shuttles, releasing, e.g., quinone-containing compounds (Lin et al., 2021). Therefore, the organic matter derived from deceased cells, also known as necromass, during the stationary growth phase could be a constant source of electron shuttles and complexing agents, favoring indirect ferrihydrite reduction.

Finally, it should be noted that Fe is a micro-nutrient essential to various cellular functions and is, therefore, routinely assimilated by bacteria, preferentially in the bioavailable Fe(II) form. Iron(III) reduction in the presence of O_2_ as a nutritional acquisition strategy has already been observed in some microorganisms like cyanobacteria (Swanner et al., 2018) when Fe(II) is scarce. Since *S. oneidensis* has an exceptionally high Fe requirement for forming essential cellular structures, particularly cytochromes (Shi et al., 2007), it is possible that they reduce Fe as a nutrient acquisition strategy. Nonetheless, the continued growth of *S. oneidensis* on sterilized spent medium without ferrihydrite (see Supplementary material—section 7) demonstrates that no Fe or other nutrient deficiency occurred over the incubation period of 72 h. For this reason, the Fe(III) reduction observed here is not likely to be a nutrient-acquisition mechanism.

### 4.2 Biomass layer thickness controls the onset of anoxic conditions

Oxygen diffuses much slower in water than in the gaseous phase. Therefore, in partially saturated sediments, water saturation and the distance between water-saturated and air-filled pores are considered crucial factors determining the formation of anoxic microsites (Chamindu Deepagoda et al., 2019; Rohe et al., 2021; Kravchenko et al., 2018; Rabot et al., 2015). However, quantitatively assessing the impact of such factors is challenging due to the difficulties of direct, *in situ* O_2_ measurements. Based on indirect measurements, Rohe et al. (2021) recently proposed that water-saturated pores more than 5 mm away from an air-filled pore could be considered anoxic. Therefore, the air-water distance and the percentage of water-filled pore space have since been considered reliable proxies of O_2_ availability (Lussich et al., 2024). In our experimental system, we could demonstrate that O_2_ diffusion into a biomass-rich layer was still highly efficient despite the presence of a 5 mm-thick water layer. Contrary to our expectations, aerobic respiration by *S. oneidensis* did not create O_2_ deficiency in the system. To quantitatively compare O_2_ diffusion versus microbial respiration and to project our observations to different water/biomass layer thicknesses and longer times, we modeled the efficiency of O_2_ diffusion in our experiment.

We simulated the O_2_ balance in our geometry using a 1D model based on Fick’s law as follows:


(1) $$\left\{ \begin{array}{c} \frac{\partial \left[O_{2}\right]}{\partial t}=D_{1}\,\frac{\partial^{2}\left[O_{2}\right]}{\partial x^{2}}\;i\,f\,x\leq s_{w\,a\,t\,e\,r}\\ \frac{\partial \left[O_{2}\right]}{\partial t}=D_{2}\,\frac{\partial^{2}\left[O_{2}\right]}{\partial x^{2}}-r\,\left(t\right)\;i\,f\,x>s_{w\,a\,t\,e\,r} \end{array}\right.$$

**FIGURE 6:**
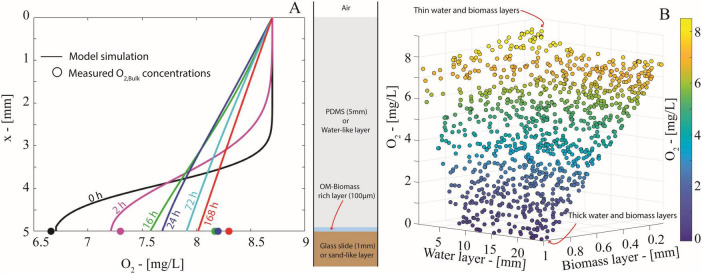
**(A)** O_2_ concentration profiles (solid lines) simulated through the mathematical model in Eq. (1) at *t* = 0, 2, 16, 24, 72, 168 h. Markers indicate the corresponding experimental values of the average O_2_ concentrations measured by the optical planar sensor at the water-solid interface. **(B)** Scatter plot displaying the O_2_ concentration at the water-glass interface at *t* = 168 h yielded by the diffusion model in Eq. (1) for 1000 randomly generated combinations of thicknesses of the water and the biomass layers.

Here, [*O*_2_], *t*, and *x* are O_2_ concentration [mg L^–1^], time [hours] and space [mm], respectively. The origin of the *x-*axis is located at the *air-water layer* interface (Figure 2B). Consequently, the solid surface is at *x* = 5 mm, as outlined in Figure 6A, and the *water-biomass layer* interface is at *x* = *s*_*water*_, i.e., 4.9 mm. The parameters *D*_1_ and *D*_2_ are the O_2_ diffusion coefficient approximated by the O_2_ diffusion coefficient in PDMS (Markov et al., 2014), i.e., *D*_1_ = 3.2 × 10^–9^ m^2^ s^–1^, and in pure water (*D*_2_ = 2 × 10^–9^ m^2^ s^–1^), respectively. Finally, the *r(t)* quantifies the aerobic respiration rate of *S. oneidensis* as a function of time, which we characterized experimentally as described in the Supplementary material (Section 8).

Air-saturated conditions (8.4 mg O_2_ L^–1^) are imposed at the *water-air layer* interface (*x* = 0 mm), with the *air layer* acting as an infinite supply of O_2_. A no-flux boundary condition is set at the *solid-water* interface (*x* = 5 mm), imposing the impermeability of the solid surface to O_2_. Further details on model implementation, initial conditions, and *S. oneidensis* respiration rate measurements are included in Supplementary material (Section 8).

Oxygen concentration profiles simulated by the model (Figure 6A) confirmed that O_2_ diffusion efficiently outpaces aerobic respiration of the growing biomass-rich layer. The simulated concentrations capture the overall increasing trend of measured O_2_ at the water-solid interface. Just after the start of the simulation (≥16 h), the O_2_ profile quickly established a linear profile, as expected in a diffusion-dominated system. The model shows that the O_2_ balance is virtually stationary after 72 h and corroborates our experimental data that a 5 mm water layer is insufficient for the onset of anoxic conditions.

To further explore the impact of water layer thickness on anoxia, we used a Monte Carlo approach (1,000 runs) to simulate the O_2_ balance with random thicknesses of the *water layer* and the *biomass layer* uniformly generated within the intervals [5, 25] mm and [0.1, 1] mm, respectively (details on random generation procedure in Supplementary material—section 9). The simulations (Figure 6B, *t* = 168 h) showed that oxic conditions persist under a combination of thin water and biomass layers, while anoxic conditions unsurprisingly develop with both thick water and biomass layers. However, biomass layer thickness had a greater impact on O_2_ concentration than the water layer thickness. A thick water layer alone did not limit O_2_ supply enough to cause anoxia. On the contrary, a thick biomass layer (>0.65 mm) reduced the O_2_ concentration by half, even with thin water layers (<5 mm). Variance-based sensitivity analysis (i.e., first-order Sobols’ indices, in Supplementary material—section 9) confirmed that over 80% of the variability in O_2_ concentration at the solid-water interface is explained by biomass layer thickness (Figure 6B). This means that the onset of anoxic conditions is not solely due to limited O_2_ diffusion imposed by the water but also depends on biomass abundance and spatial organization, which should be considered to predict anoxic microsite formation.

Our model offers key insights into microscale O_2_ dynamics, demonstrating that biomass spatial distribution should be considered for developing proxies to quantify anoxic microsite volume and determine the relative contributions of oxic and anoxic Fe(III) reduction in oxic soils and sediments. Our simulations challenge the current approach of correlating pore space saturation directly to anoxic microsite formation. Predicting anoxic microsites based on the distance between water-filled and air-filled pores and the water saturation index overestimates anoxic pore volume in partially saturated sediments, overlooking the contribution of oxic Fe(III) reduction.

Finally, in our model, both attached to solid surfaces (glass and minerals) and motile planktonic cells are assumed to contribute to O_2_ consumption. The model simulates varying organic matter-rich layer thicknesses, assuming thicker layers host more O_2_-respiring biomass, without differentiating between planktonic and attached cells. While the diffusion model offers insights into the role of aerobic biomass in anoxia onset, further investigations are needed to clarify the partitioning between attached and planktonic cells, their respective contributions to aerobic respiration, and their response to OM layer thickness for a more quantitative understanding of the anoxic microsite formation.

## 5 Conclusion

The confirmation of (direct or indirect) microbially induced ferrihydrite reduction under oxic conditions holds wide-ranging implications for the biogeochemistry of partially saturated soils and sediments regardless of the underlying biological mechanism. In addition to the contribution of anoxic microsites, microbially induced Fe(III) reduction could play a previously unrecognized role in biogeochemical element cycling in oxic sediments. Ferrihydrite, with its large specific surface area and poorly selective adsorption capacity (Dixit and Hering, 2003; Pallud et al., 2010), often acts as a sink for a variety of anions (Hu et al., 2019; Malakar et al., 2022) including hazardous metal(loid)s (e.g., arsenate, chromate, permanganate) and nutrients (e.g., phosphates). The reductive dissolution of ferrihydrite releases adsorbed anions, making it a key factor in contaminant mobility in soils (Weber et al., 2010) and groundwater systems (Li et al., 2020; Ceriotti and Guadagnini, 2021). Therefore, microbial dissolution of ferrihydrite under oxic conditions could enhance the mobility of arsenate, chromate, and other mobile metal contaminants observed in soils and shallow aquifers (Smedley, 2008; Aftabtalab et al., 2022).

It is also worth noting that our observations suggest that the use of Fe(II) as a proxy for redox conditions and anaerobiosis in partially saturated sediments and soils (Lacroix et al., 2023), is more complex than previously assumed. The process observed here, driven by *S. oneidensis*, provides a valuable starting point for further research extending these observations to diverse microbial taxa and offering new perspectives on our interpretations and the conceptual modeling of sediment biogeochemistry.

Although the oxic Fe(III) reduction rate could be significantly slower than under anoxic conditions and is yet to be estimated, its contribution might be relevant in partially saturated sediments and soils where oxic pore volume dominates occupied by anoxic microsites. Combining microfluidics with spatial sensing techniques (e.g., Zhu and Aller, 2012) provides a promising opportunity to directly investigate at the microscale how soil and sediment architecture (e.g., grain and biomass spatial arrangement, water saturation, and the connectivity of air and water-filled pores) impacts oxic/anoxic proportion and the actual contribution of anoxic microsites to Fe and other metal mobility (Ceriotti et al., 2022; Borer et al., 2018).

We highlight that, to scale the relevance of oxic Fe(III) reduction, it is imperative to determine its rate. The microfluidic experimental setup used in this study is optimized to observe microscale gradients of O_2_ but poses challenges in accurately quantifying of Fe(III) reduction rates. Future investigations should focus on quantitatively characterizing the impact of different environmental factors (e.g., carbon source and Fe(III) concentration and type).

Finally, our work provides a new perspective on possible approaches to predict anoxic microsite formation and scale the contribution of anoxic volume to Fe(III) reduction in oxic sediments and soils. For example, we envision the combination of our modeling approach to the powerful and fast-growing use of X-ray CT imaging analyses (Rohe et al., 2021) and light sheet microscopy (Ahmerkamp et al., 2022), to predict anoxic microsite formation in 3D thus overcoming the black box approaches based on indirect statistical correlations.

## Data Availability

The original contributions presented in the study are included in the article/Supplementary material, further inquiries can be directed to the corresponding author.
