# Supplementary material for: Decoupling microbial iron reduction from anoxic microsite formation in oxic sediments: a microscale investigation through microfluidic models
Source: Front Microbiol. 2025 Jan 28;16:1504111. doi: 10.3389/fmicb.2025.1504111 (PMC11811102; doi:10.3389/fmicb.2025.1504111)
Supplement: Supplementary file 1 [file Data_Sheet_1.docx]

Supplementary Material

Decoupling microbial iron reduction from anoxic microsite formation in oxic sediments: a microscale investigation through microfluidic models

Giulia Ceriotti^1†*^, Alice Bosco-Santos^1†^, Sergey M. Borisov^2^, Jasmine S. Berg^1^

^1^Institute of Earth Surface Dynamics, Faculty of Geoscience and Environment, University of Lausanne, Lausanne, Switzerland

^2^ Institute of Analytical Chemistry and Food Chemistry, Faculty of Technical Chemistry, Chemical and Process Engineering and Biotechnology, Graz University of Technology, Graz, Austria

^†^These authors share first authorship

*** Correspondence:**Giulia Ceriotti
giulia.ceriotti@polimi.it

# Microfluidic experimental setup details

## Microfluidic reactor design

We designed a straight channel shape as depicted in Figure S 1A. The chosen design was printed onto a microfluidic master using classical soft lithography in a clean room with a final thickness of 100 μm. We used the microfluidic master to mold PDMS (Sylgard 184 Silicone Elastomer mixed with 10 w/w % of curing agent; supplier: Dow Corning, Midland, MI) and replicate the straight channel as many times as needed.


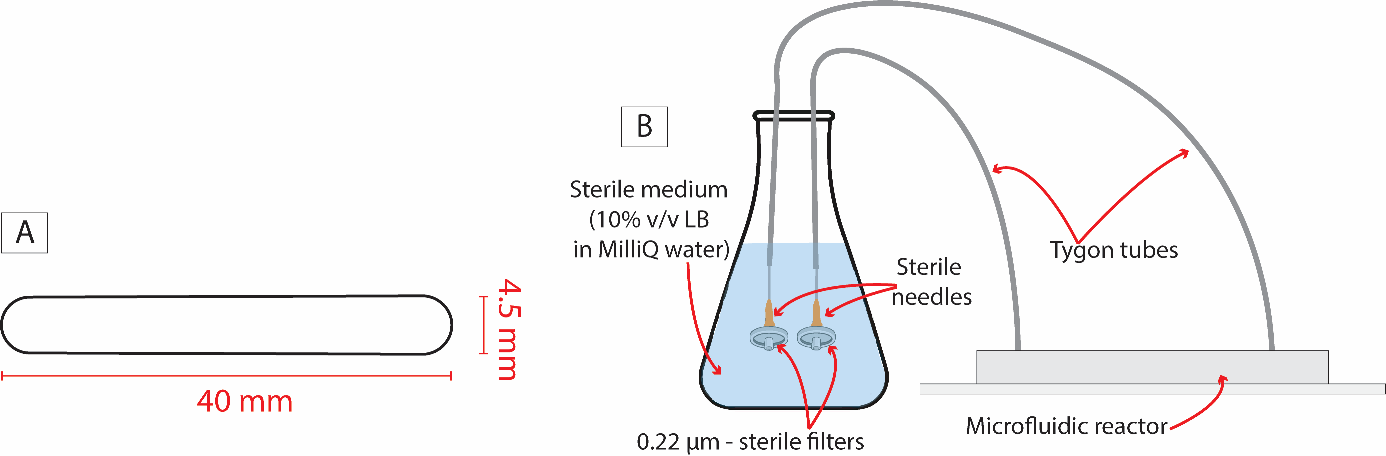


Figure S 1. A) Design of the straight channel printed onto microfluidic master; B) Outline of the setup to maintain saturation in the reactor and stagnant conditions.

The PDMS chip engraved with the straight channel shape was assembled to a glass slide using plasma bonding. When needed, the PDMS chip was bonded to a glass slide equipped with an O_2_ planar sensor, previously prepared as described in Section S2. After assembly, the microfluidic device was sterilized exposing it to UV light for 30 minutes.

The two ends of the obtained microfluidic reactor were connected to Tygon tubing (Cole-Palmer, inner/outer diameter 0.02/0.04 inches) attached to a 0.22μm- sterile filter through a needle as depicted in Figure S 1B. The two filters were immersed in an Erlenmeyer flask filled with a sterile medium (10% v/v LB broth in MilliQ water). This procedure guaranteed i) maintenance of channel saturation for very long periods, ii) maintenance of the same hydraulic potential at both ends of the microfluidic reactor to avoid any hydraulic gradient and ensure stagnant conditions, and iii) oxygen diffusion in the tubes.

Before starting the incubation, the PDMS structure was degassed under vacuum for 20 minutes. Then, a sterile syringe filled the straight channel with the experimental medium. The degassed PDMS structure absorbed any air bubbles trapped in the channel within a few minutes.

## Oxygen sensor production and calibration

The transparent O_2_ planar sensor integrated into the microfluidic reactor was obtained by screen-printing a homogeneous solution of two luminescent dyes in solid matrix polymer (polystyrene) onto a glass slide (75 mm x 25 mm) suitable for microscopy to obtain a homogenous layer with a thickness below 5 μm and shaped as a rectangle (26 mm x 4 mm). The exact composition of the coating solution was described in Ceriotti et al. (2022). The two dyes composing the sensor are excited by the same wavelength (450 nm) but show emission peaking at distinct wavelengths. The first dye, a phosphorescent Pt(II) porphyrin, emits a spectrum with a peak at 660 nm. Its peak signal intensity (I_O2_) is quenched in response to the O_2_ concentration in the fluid in contact with the sensor. The second dye, a fluorescent coumarin, has an emission spectrum that peaks at 500 nm. The peak signal intensity of coumarin dye (I_Ref_) remains unaffected by the oxygen concentration in the fluid in contact with the sensor.

The concentration of O_2_ is given as a function of the ratio R = I_O2_/ I_Ref_ so that the sensor reading is purified from possible emission fluctuations caused by factors other than O_2_ concentration variations (e.g., optical aberration, fluctuations in light intensity, etc.).

Optical configurations and video-microscopy technical details used for capturing O_2_ planar sensor signal are reported in Section S3.

Sensor calibration was performed through a two-point procedure. An air-saturated calibration solution was prepared in a Schott bottle by shaking it and exposing it to air 10 mL of Fe-enriched medium three times. A similar medium was amended with 100 mg of sodium sulfite (Na_2_SO_3_, Sigma Aldrich) to prepare the anoxic calibration solution (O_2_ concentration = 0 mg/L).

The two calibration solutions were injected into a microfluidic reactor integrated with an O_2_ planar sensor in sequence (starting with the air-saturated one). Images of the sensor luminescent signals were collected in 10 different locations along the channel length and post-processed (see Section S4 for procedures) to obtain the values of the ratio R corresponding to an O_2_ concentration of air-saturated and 0 mg/L. Oxygen concentration in the air-saturated medium was fixed at 8.35 mg/L, measured with TROX430 sensor, Pyroscience, calibrated according to the guidelines.

Following previous works, the values of O_2_ concentration and ratio R were interpolated with an exponential law using MATLAB Curve Fitting App (Ceriotti et al. 2022; Larsen et al. 2011).

$$O_{2} concentration \left[ \frac{mg}{L} \right]= 33.73e^{-2R}-2.975$$

## Comparison between the experimental designs of Ceriotti et al. (2022) and this study

This supplementary section aims to highlight similarities and differences between the experimental setups proposed in Ceriotti et al. (2022) and this study. Both studies rely on the same O_2_ sensing technology, planar sensor fabrication methods, microscope optical configuration for O_2_ sensor detection (see Section 3, Supplementary Material), O_2_ sensor image processing, and map generation. This methodological consistency ensures the reliability of our O_2_ measurements.

However, in this study, no anoxic microsites were detected while they occupied up to 2% of the space in Ceriotti et al. (2022). This different outcome can be attributed to (at least) five key differences in the microfluidic experimental design that, altogether, favored microscale O_2_ diffusion and prevented anoxic microsite formation in this study.

1. In Ceriotti et al. (2022), the external surface of the chip was coated with a gas-impermeable adhesive (NOA 81), which prevented O_2_ diffusion through the PDMS walls. This coating significantly influenced the O_2_ balance, as demonstrated by their control experiments, where anoxic microenvironment formation was negligible in uncoated chips. In contrast, our system did not use gas-impermeable coatings, allowing O2 diffusion through the PDMS material.
2. The presence of porous structure in the Ceriotti et al. system highly impacts O_2_ diffusion at the microscale. Heterogeneously arranged grains created tortuous pathways, which are well-known to increase local diffusion times. Tortuosity has indeed been identified as one of the key parameters controlling the onset of anoxic microenvironment in soils (Keiluweit et al. 2018). In contrast, our system lacks such tortuosity and therefore is less favorable to the onset of local gradients.
3. In Ceriotti et al. (2022), nutrients and electron donors were continuously supplied and rapidly distributed in the system by the pore water advection. In our system, the available nutrients and electron donors were limited to the initial medium. While the rich medium used in our system likely prevented nutrient scarcity at the macroscale, access to carbon sources at the microscale was limited by diffusion, which is less efficient for organic carbon macromolecules than for O_2_. This diffusion limitation may have slowed aerobic respiration compared to a system with a continuous supply of nutrients.
4. The flow conditions induce shear stress which can trigger bacterial responses such as extracellular polymeric substance (EPS) production and the formation of denser structures attached to solid surfaces (Ramasamy and Zhang 2005). The result is a very heterogeneous distribution of biomass, organized in compacted colonies of various shapes, as observed in Ceriotti et al. (2022) and elsewhere (Carrel et al. 2018). Our stagnant system lacked shear stress and, consistently, we observed the formation of a homogeneously distributed and less compacted biomass layer. This difference in biomass structure could also explain why our stagnant system was less prone to the formation of localized O_2_ gradients.
5. Ceriotti et al. (2022) used *P. putida* GB1 while this study focuses on *S. oneidensis* MR-1. Given their different metabolic capabilities, these two strains will likely present different oxygen consumption rates. Although a detailed comparison of the O_2_ consumption rates of these strains is beyond the scope of the present work, these physiological differences could influence the oxygen consumption rates and the establishment of microscale gradients.

# Ferrihydrite synthesis

Ferrihydrite was synthesized by reacting 1 L of 0.1 M Fe(NO₃)₃·9H₂O with 500 mL of 1 M KOH under sterile conditions. Both solutions were filtered through 0.2 µm filters before use. Under a laminar flow hood, the Fe(NO₃)₃·9H₂O solution was stirred in a sterile 2 L bottle while the pH was monitored using a cleaned and sterilized pH electrode. The KOH solution was added gradually (1 mL at a time), with stirring, until the pH reached 7.5–8.0, typically requiring 400–600 mL of KOH. When approaching the desired pH, KOH was added dropwise, allowing the solution to equilibrate between additions.

The resulting precipitate was collected by centrifugation (500 mL vessels, maximum speed, 20 minutes) and resuspended in 50 mL falcon tubes. The pellets were rinsed three times with 20 mL of filtered HEPES buffer (pH 8.0). The ferrihydrite was then freeze-dried overnight, ground into a fine powder with a flame-sterilized mortar and pestle (approximately 1 hour), and weighed in a pre-weighed 50 mL falcon tube to determine the total yield. This procedure typically produced approximately 10 g of ferrihydrite per liter of Fe(NO₃)₃·9H₂O solution.

# Video-microscopy setup and optical configuration

Video-microscopy was performed with an inverted automated scope Eclipse Ti-E2, Nikon, controlled by NIS-Element software and equipped with i) a 10X objective; and ii) a CMOS DS-Qi2 (Nikon) with a sensor area of 36.0 mm x 23.9 mm and an actual pixel size of 7.3 μm and exposure time fixed at 200 ms. Images were taken at 10 locations every hour along the channel longitudinal direction, stitching together 4 pictures at each location to capture a more extensive area obtaining a final size of 9871 x 6365 pixels (corresponding to an area of ~ 5.2 mm^2^) for each image.

The scope automatically switched between 4 different optical configurations:

- **Bright Field (BF).** This optical configuration used a diascopic scheme illuminating the device with the white LED light source at 0.5% of its maximum intensity. Opaque elements like ferrihydrite minerals appeared as dark objects on a white background.
- **Adjusted Phase Contrast (a-PC)**. The same illumination source employed in BF was used at 64.3% of its maximum intensity combined with a Nikon Ph3 phase contrast plate, compared to the ring (a Ph1) present in the selected objective. This optical configuration detected all elements with an optical density different from the liquid medium, making ferrihydrite minerals and microbial cells appear as bright objects on a dark background.
- **Fluorescence at 500 nm (F_500_) and 660 nm (F_660_)**. These optical configurations used an episcopic illumination scheme to capture the signals of the reference and O_2_-sensitive dyes, which make up the O_2_ planar sensor. A blue LED (440 ± 20 nm, Lumencor SPECTRA X Light Engine) illuminated the microfluidic reactor, exciting the luminescent dyes of the O_2_ planar sensor. The luminescent signal is filtered by Semrock bandpass emission filters (500 ± 20 nm for the reference and 650 ± 13 for the O_2_ sensitive signals).

# Image processing and computation of bulk parameters

Image processing was performed in the MATLAB® environment (R2021b, version 9.11.0.1769968) with in-house produced codes.

**Ferrihydrite spatial organization.** Images captured with BF configuration were normalized to the highest pixel value recorded in each image, so each pixel value ranged between 0 and 1. The probability density function (*pdf)* of pixel value distribution and its 10^th^ percentile (p_10_) were computed. Pixels with values smaller than p_10_ were identified as dark objects, i.e., ferrihydrite minerals. A binary matrix (*FE_OX*) of the same size as the original BF image was created for each acquisition time to store the spatial organization of ferrihydrite minerals assigned a value of 0 and all others assigned a value of 1.

**Biomass spatial organization and bulk microbial growth.** Images collected with the a-PC configuration were normalized to the maximum pixel value recorded in each image, resulting in pixel values between 0 and 1. Higher pixel values corresponded to objects with higher optical density than the liquid medium, such as microbial cells and minerals. These objects were identified by setting a threshold at 0.3 (chosen on preliminary assessment using images collected at t = 0 hours) and forcing all pixel values smaller than 0.3 to 0. To distinguish microorganisms from minerals, we multiplied the threshold a-PC image by the *FE_OX* matrix associated with the exact location and acquisition time. In the resulting map (*BIO*), only pixels associated with biomass presence showed values larger than 0.

Bulk microbial growth (*BG_Bulk_*) was computed for each acquisition time (*t*) by processing the corresponding matrix *BIO* as follows

$${BG}_{Bulk}(t)=\frac{\sum_{i=1}^{N} BIO(i, t)}{BG\_0 \sum_{i=1}^{N} FE\_OX(i, t)}$$

where *i* is the image pixel counter, *N* is the total number of pixels of the image, and *BG_Bulk_* (*t*=0) is equal to

$$BG\_0=\frac{\sum_{i=1}^{N} BIO(i, t=0)}{\sum_{i=1}^{N} FE\_OX(i, t=0)}$$

**O_2_ concentration maps and bulk O_2_ concentration.** Spatial distributions of the ratio R were computed by dividing the images collected with F_660_ configuration by those collected with F_500_ one, pixel by pixel. O_2_ concentration maps (*OX*) were obtained by applying the planar sensor calibration curve equation to each pixel value of R spatial maps.

Bulk O_2_ concentration (*O_2,Bulk_(t)*) was computed for each acquisition time (*t*) by processing the corresponding matrix *OX* as follows

$$O_{2,Bulk}(t)=\frac{\sum_{i=1}^{N} OX(i, t)}{N}$$

# Ferrozine assay

A sample of > 70 μL was obtained by extracting and mixing the medium incubated in 5 replicates of the microfluidic reactor and treated in the following way:

- An aliquot of 80.6 μL of 0.1 M HCl was added to 70 μL of the sample in a 1.5 mL Eppendorf centrifuge conical tube to lyse bacterial cells and desorb Fe(II) from mineral surfaces during a 15 min reaction time.
- The solution was centrifuged for 15 minutes at 12000 rpm in a microcentrifuge to separate solids from the liquid phase.
- An aliquot of 140 μL of the supernatant is pipetted into a micro-well of a Greiner Microplate (96 wells, PS, F-bottom, clear) and mixed with 20 μL of Ferrozine reagent.
- After 10 minutes of reaction, the sample absorbance at 560 nm (ABS_560_) was measured using a Spark® Multimode Microplate Reader.
- The absorbance measurements are translated into Fe(II) concentrations by applying the following calibration curve

$$Fe\left( II \right) concentration \left[ mM \right]=\left\{ \begin{aligned} 2.68{ABS}_{560}-0.122 if {ABS}_{560}\leq0.1 \\ 1.52{ABS}_{560}-0.014 if{ABS}_{560}>0.1 \end{aligned} \right.$$

The calibration curve was obtained by interpolating the absorbance measured for 8 calibration solutions (Fe(II) concentrations = 0, 0.005, 0.01, 0.015, 0.03, 0.1, 0.2, 0.5 mM) obtained by dissolving Fe-sulfate into the sterile medium, processed according to the same protocol applied to the samples. As O_2_ can oxidize Fe(II), the determination of its concentrations might be underestimated (Porsch and Kappler 2011; Posner 1953).

# Definition of anoxic microsite percentage and its detection limit

To assess the formation of anoxic microsites, each O_2_ concentration map (*OX*) was characterized in terms of the percentage of anoxic surface (*P_AS_* [%]). In this work, we assumed to be *anoxic* the space characterized by an O_2_ concentration ≤ 0.32 mg/L, a threshold traditionally associated with the onset of microoxic conditions(Berg et al. 2022). To this end, we generated a binary matrix (*ANOX*) with the same size as the O_2_ concentration map as follows

$$ANOX\left( i \right)= \left\{ \begin{aligned} 1 if OX\left( i \right)\leq0.32 \frac{mg}{L} \\ 0 if OX\left( i \right)>0.32 \frac{mg}{L} \end{aligned} for i=1,\ldots,N \right.$$

where *i* is the pixel counter, and *N* is the total number of pixels of the O_2_ concentration map. The resulting matrix *ANOX* identified all the pixels where the onset of anoxic conditions was observed (i.e., with O_2_ concentration ≤ 0.32 mg/L). The percentage of anoxic volume results from

$$P_{AS}(t)=\frac{\sum_{i=1}^{N} ANOX(i, t)}{\sum_{i=1}^{N} OX(i, t)}100$$

Small impurities entrapped in the microfluidic reactor, burnt pixels of the camera sensor, and imperfections of the O_2_ planar sensor surface may locally interfere with the reading of the O_2_ concentration and generate background noise in the computation of *P_AS_*. To estimate the order of magnitude of the *P_AS_* background noise, we saturated a microfluidic reactor equipped with the O_2_ planar sensor with a sterile air-saturated medium solution (20 mM PIPES, 2 mM ferrihydrite and 10X LB with O_2_ concentration = 8.4 mg/L). To prepare the air-saturated solution, we used the same procedure reported in Section S2. Images of the sensor luminescent signals were collected in 10 different locations along the longitudinal direction of the channel and post-processed to obtain the corresponding O_2_ concentration maps, as described in Section S4. We computed the corresponding *P_AS_* by applying the procedure reported above. Results are reported in Table S1, along with their mean value.

Table S 1 PAS values computed for an air-saturated sterile medium in 10 different locations and their mean value.

| Image | 1 | 2 | 3 | 4 | 5 | 6 | 7 | 8 | 9 | 10 | Mean Value |
| --- | --- | --- | --- | --- | --- | --- | --- | --- | --- | --- | --- |
| P_AS_[%] | 1.1x10^-4^ | 2.0x10^-4^ | 7.1x10^-6^ | 1.4x10^-5^ | 0 | 7.6 x10^-3^ | 2.1x10^-3^ | 1.8x10^-4^ | 0 | 0 | 1.0x10^-3^ |

Based on this assessment, we defined the value of *P_AS_* = 1.0 x 10^-3^ as a technical detection limit for identifying anoxic micro-site formation. Any value of P_AS_ falling below the detection limit was considered indistinguishable from the background noise and, therefore, was not related to the actual formation of anoxic conditions.

# Impact of Fe and other nutritional limitations in LB after 72 hour of aerobic growth

In this experiment, we aimed to investigate the nutritional and, specifically, Fe limitations for the growth of *S. oneidensis* under aerobic conditions. The experiments were conducted in a batch reactor kept in the dark under continuous stirring to ensure oxic and well-mixed conditions using the same experimental medium injected in the microfluidic reactor without ferrihydrite enrichment (1:10 v/v Luria Bertani broth in deionized water buffered by 20 mM PIPES). The overall incubation lasted 145 hours. S. oneidensis was inoculated from overnight culture (1:50 v/v), similarly to the microfluidic setup. After 72 h of incubation, the medium was filtered through a 0.22 μm filter. Subsequently, a fresh inoculum of Shewanella oneidensis was introduced into the sterile-filtered medium. Soluble Fe(II) was monitored at 24-h intervals (Figure S 2A) using ferrozine assay. Simultaneously, the protein content, assessed by the Coomassie assay, was used as a proxy for bacterial growth (Figure S 2B). The persistence of oxic conditions in the reactors was constantly monitored through O_2_ sensor spots of 5 mm diameter (OXSP5 supplied by PyroScience). The observation revealed that *S. oneidensis* demonstrated renewed growth in the spent medium after filtration, suggesting the absence of nutritional limitation and toxicity by LB decomposition byproduct in the chosen medium.


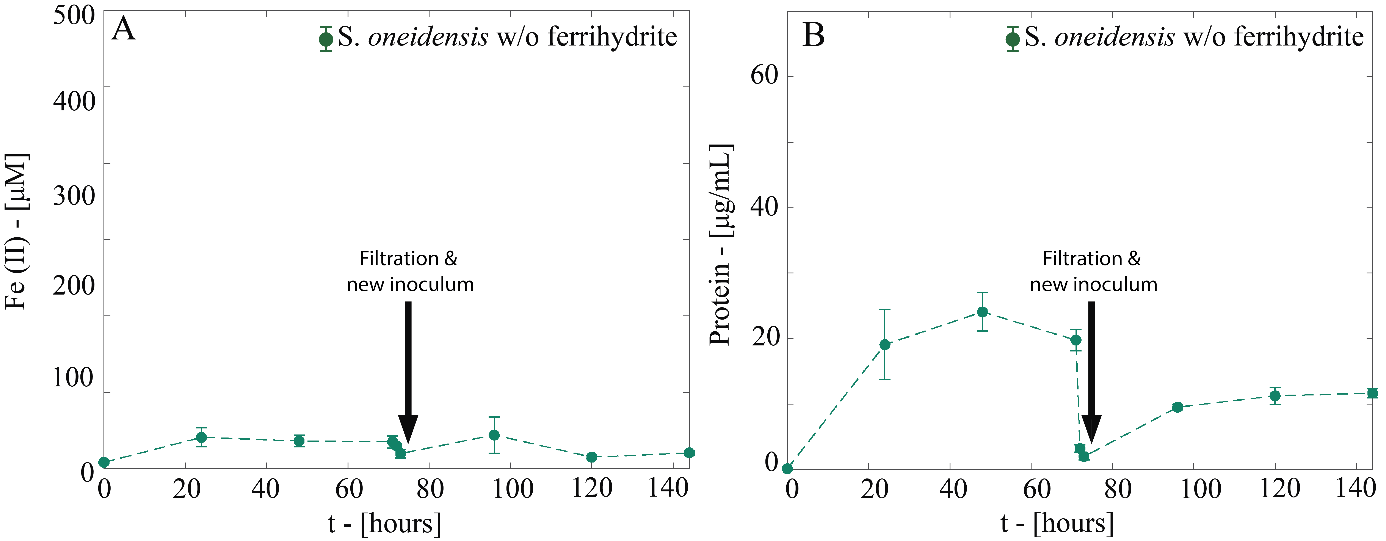


Figure S 2 - Shewanella oneidensis growth in LB buffered medium under aerobic conditions. Fe(II) concentrations (A) and protein content (B) were monitored over 145 h in the dark with continuous stirring. Inoculation at 0h, followed by sterile filtration and re-inoculation after 72 h, allowed renewed bacterial growth, indicating the absence of nutritional limitations in the culture medium.

# Diffusion model

The model comprises one partial differential equation whose formulation changes as a function of space.

The model was numerically solved using the function *pdepe* of MATLAB® (R2021b, version 9.11.0.1769968). The *pdepe* function automatically adjusted the time mesh discretization to meet the integration tolerance, and the space mesh (*x*) discretization was set equal to 0.01 mm.

We set the O_2_ concentration at air-saturation level as a boundary condition at *x* = 0, i.e., representing the *water-air layer* interface, and imposed no flux at the solid surface (*x* = 5 mm). The initial concentration in the biomass layer was assumed to match the first O_2_,_bulk_ measurement yielded by O_2_ maps (6.6 mg/L). For the *water layer*, the initial conditions are unknown. Reasonably, we assume that O_2_ concentration initially resembled air saturation. However, the lower initial O_2_ concentrations observed in the biomass layer likely affected the part of the *water layer* in direct contact with the *biomass layer*. To impose such initial conditions in the *water layer*, we imposed the following continuous function:

$$\left[ O_{2} \right]\left( t=0 \right)= -1.05\left[ \frac{1+\mathrm{erf} \left( x-\mu\right)}{\sigma\sqrt{2}} \right]+8.7 for 0<x<4.9 mm$$

where μ = 3.9 mm and σ = 00.5 x 10^-3^ mm. As a result, the *water layer* showed an initial O_2_ concentration like the initial condition of the *biomass layer* only in its proximity*.* For distances larger than 1 mm from the *biomass layer, the* initial O_2_ concentration rapidly approached the air-saturation concentration*.*

In the *water layer* (PDMS), bacteria are not present. Therefore, the only active process on O_2_ balance is diffusion. In the *biomass layer*, bacteria respire aerobically and are modeled as a sink of O_2_ between 4.9 mm and 5 mm. The sink term appears in Eq (1) of the manuscript as *r(t)* [mg L^-1^ s^-1^], and it is time-dependent because of biomass density increment in the system with time. Also, it is reasonable to expect that biomass respires at a higher rate during the fast-growing phase than in the stationary one.

We experimentally estimated the *r(t)* value following the procedure proposed by Warkentin et al. (2007). *S. oneidensis* was grown aerobically in an Erlenmeyer flask using the same sterile medium used in the microfluidic reactor without ferrihydrite. At different sampling times, a 1.5 mL aliquot of the bacterial culture was transferred in a short thread vial (32 x 11.6 mm, BGB, Germany) integrated with an O_2_ spot sensor (TROX430, Pyroscience), as outlined in Figure S 3A. The spot sensor was calibrated according to the supplier's guidelines before use. Oxygen concentrations were monitored every 20 minutes using the Pyroscience workbench software. This procedure was repeated three times for samples taken from independent bacterial cultures for all sampled times. The O_2_ concentration always attained a linear trend for all replicates with R^2^ larger than 0.98. The fitted slope indicated the O_2_ consumption rate *r(t)*. The experimental values of *r(t)* (Figure S 3B) were interpolated to estimate *r(t)* for each time mesh used by the *pdepe* numerical solver.


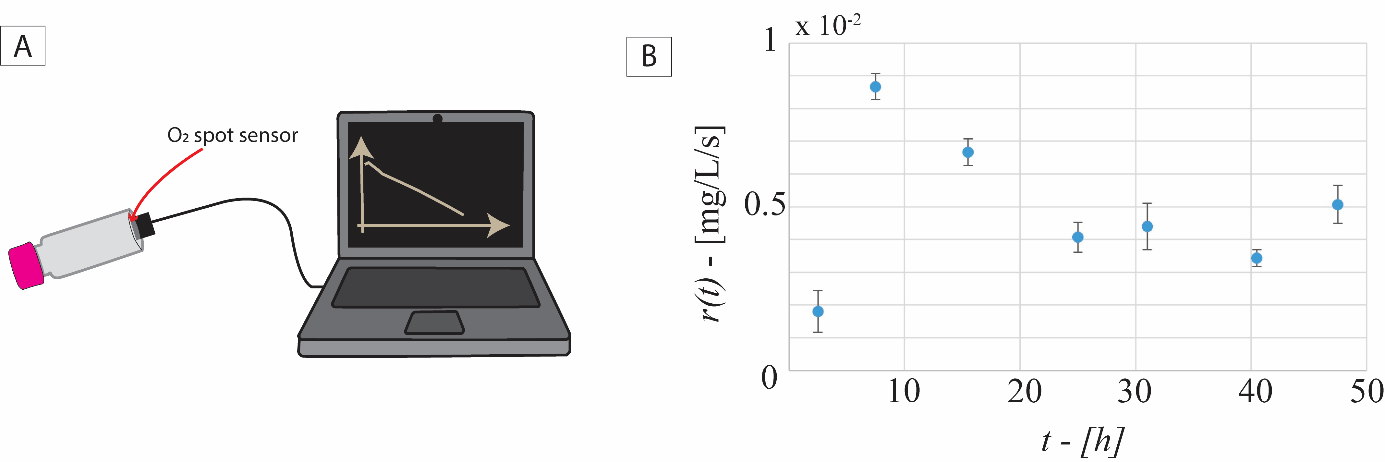


Figure S 3 – A) Outline of the experimental setup used to measure the O_2_ respiration rate (r(t)) at different times during its growth. B) Mean values of r(t) measured at different times and the associated standard deviation.

# Variance-based sensitivity analysis

A thousand random couples of *biomass* and *water layer* thickness values were generated from the probability density functions with uniform distributions in [0.1, 1] and [5, 25] mm, respectively. The script for random value generation was prepared in MATLAB® (R2021b, version 9.11.0.1769968). Using a Monte Carlo approach, the diffusion model in Eq. (1) was run for each random couple of thickness values, and the O_2_ concentration simulated at the solid interface after 168 hours was the target output variable used for the variance-based sensitivity analysis.

Sobols’ indices are well-known variance-based sensitivity metrics widely implemented in many fields of environmental and Earth sciences (Ceriotti et al. 2018). Such indices are used to investigate the relative contribution of model parameters, alone or combined, to the variance of the target output variable. In other words, these indices quantify how sensitive the output target variable is to each parameter alone or combined with the others. In this work, we used the first-order indices defined as

$$S_{j}= \frac{Var[E[Y|P_{j}]]}{Var[Y]}$$

Here, *Y* is the target output variable, i.e., O_2_ concentration at the solid surface after 168 hours of simulation. *P_j_* is the *j*-th random parameter of the model included in the sensitivity analysis. In this study, we consider two parameters: *water layer* thickness (*P_1_*) and *biomass layer* thickness (*P_2_*). The symbol *E*[*Y*|*P_j_*] represents the mean value of *Y* conditional to the parameter *j-*th, and *Var*[] refers to the variance of the quantity included in the brackets.

First-order Sobols’ indices quantify how much the *j*-th parameter contributes to the variance of the output variable alone(Saltelli et al. 2008). First-order Sobols’ indices for the *water* and *biomass layer* thickness are equal to 0.2 and 0.8, respectively.

# References

Berg, Jasmine S, Soeren Ahmerkamp, Petra Pjevac, Bela Hausmann, Jana Milucka, and Marcel MM Kuypers. 2022. "How low can they go? Aerobic respiration by microorganisms under apparent anoxia." *FEMS Microbiology Reviews* 46 (3):fuac006.

Carrel, Maxence, Verónica L Morales, Mario A Beltran, Nicolas Derlon, Rolf Kaufmann, Eberhard Morgenroth, and Markus Holzner. 2018. "Biofilms in 3D porous media: Delineating the influence of the pore network geometry, flow and mass transfer on biofilm development." *Water research* 134:280-291.

Ceriotti, G, L Guadagnini, G Porta, and A Guadagnini. 2018. "Local and global sensitivity analysis of Cr (VI) geogenic leakage under uncertain environmental conditions." *Water Resources Research* 54 (8):5785-5802.

Ceriotti, Giulia, Sergey M Borisov, Jasmine S Berg, and Pietro de Anna. 2022. "Morphology and size of bacterial colonies control anoxic microenvironment formation in porous media." *Environmental Science & Technology* 56 (23):17471-17480.

Keiluweit, Marco, Kaitlyn Gee, Amanda Denney, and Scott Fendorf. 2018. "Anoxic microsites in upland soils dominantly controlled by clay content." *Soil Biology and Biochemistry* 118:42-50.

Larsen, Morten, Sergey M Borisov, Björn Grunwald, Ingo Klimant, and Ronnie N Glud. 2011. "A simple and inexpensive high resolution color ratiometric planar optode imaging approach: application to oxygen and pH sensing." *Limnology and Oceanography: Methods* 9 (9):348-360.

Porsch, Katharina, and Andreas Kappler. 2011. "FeII oxidation by molecular O2 during HCl extraction." *Environmental Chemistry* 8 (2):190-197.

Posner, AM. 1953. "The kinetics of the charcoal catalyzed autoxidation of Fe 2+ ion in dilute HCl solutions." *Transactions of the Faraday Society* 49:389-395.

Ramasamy, Parashuram, and X Zhang. 2005. "Effects of shear stress on the secretion of extracellular polymeric substances in biofilms." *Water Science and Technology* 52 (7):217-223.

Saltelli, Andrea, Marco Ratto, Terry Andres, Francesca Campolongo, Jessica Cariboni, Debora Gatelli, Michaela Saisana, and Stefano Tarantola. 2008. *Global sensitivity analysis: the primer*: John Wiley & Sons.
